# Raspberry-like Nævus

**Published:** 1847-04

**Authors:** 


*Raspberry-like Nævus.*—Mr. Thompson, of Stalybridge, presented four nævi, removed by ligature, which were interesting by reason of the proof they afforded of their hereditary character. One was larger than a walnut, and three equalled raspberries in form and size. In colour they were bright blood red, and their surfaces resembled exactly the raspberry in appearance.

They were removed from the right shoulder of a very healthy woman, aged 44 years, who had borne eight children. She first noticed the large nævus in her eighteenth year. It was then like an ordinary brown mole, possessing very little sensibility. After a time it increased in size, and at length became very sensitive and painful, and acquired the appearance described. It was attached to the skin by a peduncle about a quarter of an inch in diameter, which was occasionally excoriated, and discharged a very offensive fluid.

This woman's daughter, maternal grandmother, and great grandfather, have all had similar nævi.—*Ibid.*

